# Potential Role of Exercise Induced Extracellular Vesicles in Prostate Cancer Suppression

**DOI:** 10.3389/fonc.2021.746040

**Published:** 2021-09-14

**Authors:** Ying Zhang, Jin-Soo Kim, Tian-Zhen Wang, Robert U. Newton, Daniel A. Galvão, Robert A. Gardiner, Michelle M. Hill, Dennis R. Taaffe

**Affiliations:** ^1^Department of Physiology, Harbin Medical University, Harbin, China; ^2^Exercise Medicine Research Institute, Edith Cowan University, Joondalup, WA, Australia; ^3^School of Medical and Health Sciences, Edith Cowan University, Joondalup, WA, Australia; ^4^Department of Pathology, Harbin Medical University, Harbin, China; ^5^School of Human Movement and Nutrition Sciences, University of Queensland, Brisbane, QLD, Australia; ^6^UQ Centre for Clinical Research, University of Queensland, Brisbane, QLD, Australia; ^7^Department of Urology, Royal Brisbane and Women’s Hospital, Brisbane, QLD, Australia; ^8^QIMR Berghofer Medical Research Institute, Brisbane, QLD, Australia

**Keywords:** prostate cancer, exercise oncology, extracellular vesicles, cancer physiology, exercise physiology

## Abstract

Physical exercise is increasingly recognized as a valuable treatment strategy in managing prostate cancer, not only enhancing supportive care but potentially influencing disease outcomes. However, there are limited studies investigating mechanisms of the tumor-suppressive effect of exercise. Recently, extracellular vesicles (EVs) have been recognized as a therapeutic target for cancer as tumor-derived EVs have the potential to promote metastatic capacity by transferring oncogenic proteins, integrins, and microRNAs to other cells and EVs are also involved in developing drug resistance. Skeletal muscle has been identified as an endocrine organ, releasing EVs into the circulation, and levels of EV-containing factors have been shown to increase in response to exercise. Moreover, preclinical studies have demonstrated the tumor-suppressive effect of protein and microRNA contents in skeletal muscle-derived EVs in various cancers, including prostate cancer. Here we review current knowledge of the tumor-derived EVs in prostate cancer progression and metastasis, the role of exercise in skeletal muscle-derived EVs circulating levels and the alteration of their contents, and the potential tumor-suppressive effect of skeletal muscle-derived EV contents in prostate cancer. In addition, we review the proposed mechanism of exercise in the uptake of skeletal muscle-derived EVs in prostate cancer.

## Introduction

Prostate cancer (PCa) is the most frequently diagnosed cancer in 112 countries with over 1.4 million new cases estimated in 2020, which is 14.1% of all new cancer diagnoses (1). Moreover, 370,000 men were estimated to die from prostate cancer in 2020 (6.8% of deaths caused by all cancer) (1). Early detection and advancement in treatments have improved survival for patients with PCa (1). However, these treatments can also have enduring adverse effects, such as the loss of lean mass and bone mass, fat mass gain, post-surgery incontinence, metabolic imbalance, and reduced quality of life (2–4).

Exercise or physical activity has been receiving attention in patient care in the oncology setting (5) due to the increasing body of research in the field of exercise oncology. Multiple epidemiological studies (6, 7) and clinical trials (8–10) consistently report improvements in health-related outcomes for PCa patients. In addition, preclinical murine model studies have also demonstrated a reduced PCa tumor volume and delayed tumor growth with an exercise stimulus (11, 12), and provide a strong mechanistic case for clinical trials to be tested on cancer outcomes. However, while numerous hypotheses exist, the mechanisms by which exercise influences tumor biology are not fully understood (13).

As such, multiple studies have been conducted to reveal the mechanisms of exercise-induced benefits for cancer patients. Alteration in circulating factors, epigenetic modulation, gene expression modulation, immune function improvement, and systemic inflammation reduction have been suggested as potential mechanisms for exercise-induced tumor suppression (13, 14). For instance, serum levels of myokines, skeletal muscle secreted cytokines and peptides, are known to be altered with skeletal muscle stimulation, and multiple preclinical studies have shown the tumor-suppressive role of myokines with direct application in different cancer cell lines, including PCa (13). Although the beneficial role of exercise in reduced disease progression, increased survival, and patient care is promising, the mechanisms underlying how exercise-induced physiological changes provide tumor-suppressive effects are not clearly understood.

One potential mechanism proposed is the involvement of extracellular vesicles (EVs) (15, 16), as exercise-induced skeletal muscle-derived EVs may reduce cancer cell proliferation and metastasis (17). EVs are small membrane-surrounded structures released from various cells (18) that transfer bioactive molecules (including DNA, RNA, and proteins) from donor to acceptor cells (19). Two main types of EVs are defined based on their cellular route of release, exosomes and microvesicles or microparticles (18). The term ‘exosome’ refers to vesicles of the endosomal system that are released through the fusion of the multivesicular body delimiting membrane with the plasma membrane, while ‘microvesicles’ or ‘microparticles’ refer to vesicles that directly pinch off the cell surface (18).

During exercise, the release of EVs packaging cytokines and myokines plays a crucial role in the communication between muscle and other tissues (20). For instance, the skeletal muscle-derived EVs have been shown to increase in response to exercise, and increased uptake of skeletal muscle-derived EVs in the liver has been shown in animal models (21). Thus, the potential role of skeletal muscle-derived EVs in reducing cancer proliferation and migration by transporting anti-oncogenic proteins and microRNAs (miRNAs) has been proposed (20). This review will provide the current evidence for the role of exercise in skeletal muscle-derived EVs concentration in the circulatory system, the alteration of EV contents, and the potential role of exercise-induced skeletal muscle-derived EVs content in PCa. In addition, we propose a potential mechanism whereby exercise enhances skeletal muscle-derived EV uptake and delivery in PCa.

## Extracellular Vesicles in Prostate Cancer

**Figure 1:**
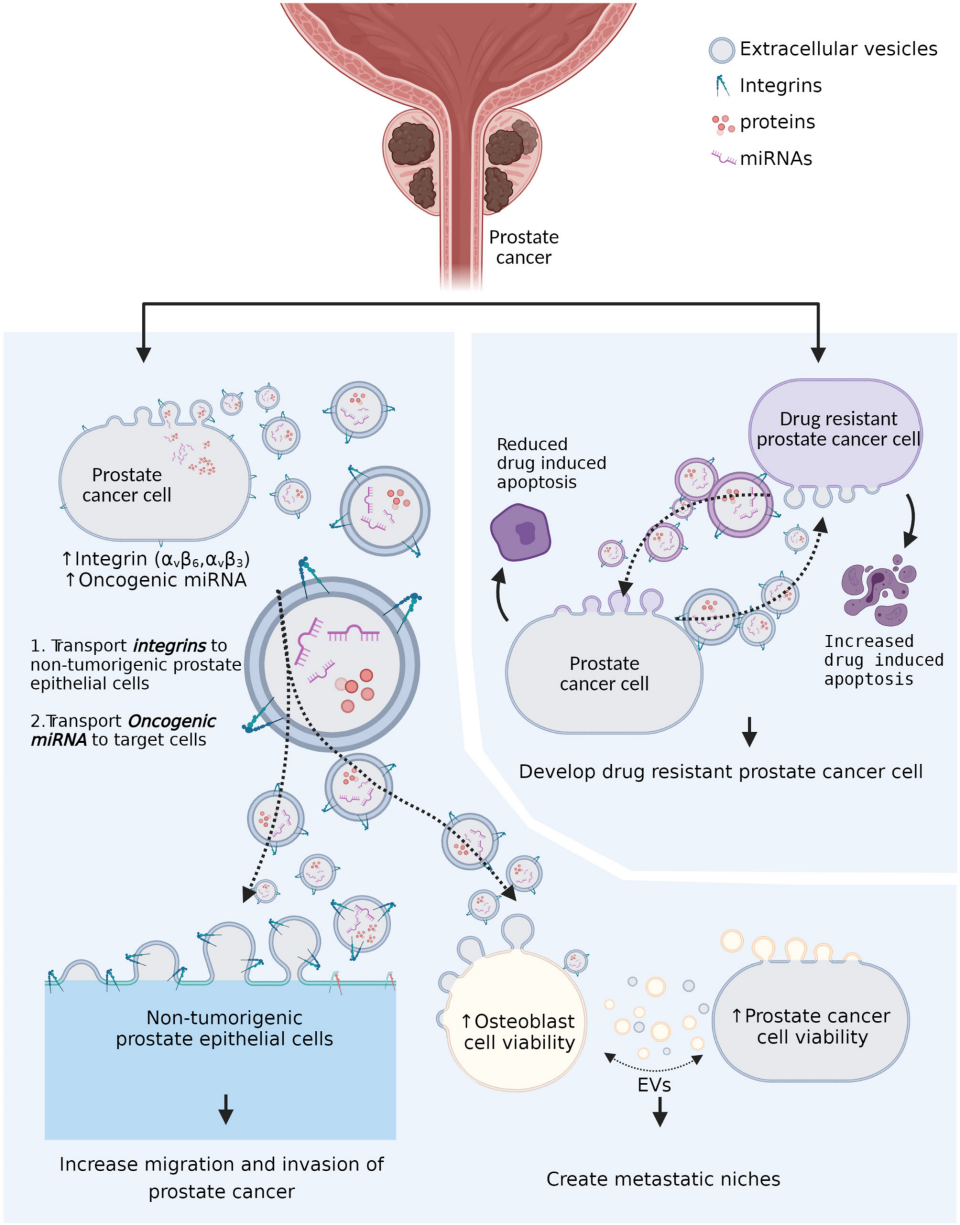
The role of tumor-derived extracellular vesicles in prostate cancer progression and drug resistance. Tumor-derived extracellular vesicles (EVs) transport the integrins (α_V_ family integrins) to non-α_V_ family integrin presenting cells and promote cell-extracellular matrix communication to promote cancer growth. Tumor-derived extracellular vesicles also transport oncogenic microRNAs (miRNAs) to recipient cells and promote remodeling of metastatic niches. In addition, drug resistant prostate cancer cell-derived extracellular vesicles can reduce drug-induced apoptosis in non-drug resistant prostate cancer cells. Moreover, non-drug-resistant prostate cancer cell-derived extracellular vesicles can increase drug-induced apoptosis in drug-resistant prostate cancer cells.

EV cargoes are considered to be biologically influential in cancer progression, metastasis, and development of drug resistance (22–24). Furthermore, particular miRNAs in EV cargoes have been considered as potential diagnostic, prognostic, and predictive markers for PCa (24–26) (**Figure 1**). For instance, miR-107, miR-130b, miR-141, miR-2110, miR-301a, miR-326, miR-331-3p, miR-432, miR-484, miR-574-3p, and miR-625 were shown to be substantially increased in circulating EVs from prostate cancer patients (n=78) compared to healthy individuals (n=28) (P<0.05) (27). Out of 11 miRNAs shown to increase in prostate cancer patients, miR-141 and miR-375 were significantly increased in patients with metastatic prostate cancer (n=16) compared to patients with localized prostate cancer (n=55) (P<0.05) (27), suggesting potential of circulating exosomes as prognostic markers for PCa. Furthermore, exosomal RNA analysis of plasma from 100 castration-resistant prostate cancer (CRPC) patients showed significantly shorter overall survival in patients with higher miR-375 and miR-1290 levels compared to those with lower miR-375 and miR-1290 levels (P=0.0045) (28). Although further validation will be required, this study also showed improved performance of predictive models for overall survival by incorporating miR-375 and miR-1290 levels with clinical prognostic factors (time to ADT failure and PSA level at the time of CRPC diagnosis) (28).

The study by Albino and colleagues (29) investigating the role of circulating miR-424 positive EVs in PCa patients demonstrated that metastatic PCa patients (metastatic castration-sensitive (mCSPC), n=16; metastatic castration-resistance (mCRPC), n=17) showed a higher frequency (*P*<0.05) of circulating miR-424 positive EVs compared to patients with primary tumors (n=25) and benign prostatic hyperplasia (BPH, n=6). Moreover, the application of EVs isolated from plasma of patients (n=17) in the *in vitro* environment showed increased tumor-sphere formation in the application of EVs from patients with mCSPC and mCRPC compared to primary or BPH patients (*P*<0.02) (29). In addition, the level of miR-424 containing EVs was positively associated with tumor growth in the 3D cell culture environment (tumor-sphere formation) (*P*=0.003) (29), suggesting a potential role of EV-contained miRNAs in PCa progression.

The study by Albino et al. (29) also generated a castration-resistant cell model using the LNCaP cell line by culturing in an androgen-depleted condition and showed significantly increased miR-424 in castration-resistant LNCaP-derived EVs compared to EVs derived from normal LNCaP cells (*P*<0.005), confirming the elevation in circulating miR-424 positive EVs in patients with advanced PCa (29). Furthermore, the application of EVs isolated from the castrate-resistant LNCaP-derived EVs to another PCa cell line, RWPE-1, showed increased tumor formation in a 3D cell culture model and cell migration compared to the application of normal LNCaP cell-derived EVs (*P*<0.005) (29), confirming the result by applying human plasma isolated EVs to cancer cell lines.

Preclinical studies involving PCa cell line-derived EVs and osteoblast cells also suggest a role for cancer cell-derived EVs in creating favorable niches for metastasis (30–32). In an *in vitro* study investigating the role of prostate cancer cell-induced EVs in osteoclastogensis and osteoblast proliferation, Inder and colleagues (30) demonstrated an increase in osteoclastogenesis of murine RAW264.7 pre-osteoclast cells (37 fold) and human primary-osteoblast proliferation (1.43 fold) with the presence of EVs isolated from the human prostate cancer cell line PC3 compared with no PC3- induced EVs (P<0.005). However, secreted soluble factors from PC3 cells were not able to increase osteoclastogensis of RAW264.7 cells, indicating requirement for PCa-induced EVs in osteoclastogenesis (30).

Probert and colleagues (31) reported that culture of osteoblast cells with PC-3 (high metastasis capacity to bone), C4-2 (moderate metastasis capacity to bone), and C4-2-4B (a bone metastatic lineage of C4-2) PCa cell line-derived EVs resulted in a significant increase of osteoblast cell viability (*P*=0.004, *P*=0.032, and *P*=0.001, respectively). In addition, co-culture of the osteoblast cells preincubated with PCa derived-EVs with PC-3 and C4-2 cells showed a significant increase (*P*<0.001) in PCa cell line viability compared to PCa cell lines co-cultured with osteoblast cells precultured with non- PCa cell lines (31). This study also showed a significant increase of PCa abundant miRNA in osteoblast and induced functional changes of osteoblast *via* EVs transported miRNA, suggesting a role of PCa cell line-derived EVs in generating metastatic niches (31).

Similarly, there is also preclinical evidence for the role of EVs in transferring integrins to different subsets of PCa cell lines (33, 34). The integrins are a diverse family of glycoproteins that allows cells to interact with extracellular matrix (ECM) molecules and, in cancer, overexpression of the integrins in cancer cells contributes to migration and invasion by disrupting the ECM molecules (35). Studies have showed increased integrins (such as α_V_β_6_ and α_V_β_3_) in PCa cell lines (PC-3 and RWPE) and PCa cell line-derived exosomes (33, 34). Incubation of α_V_β_6_ negative PCa cell line (DU145) or non-tumorigenic prostate epithelial cells with PCa cell line-derived exosomes results in *de novo* expression of these integrins in α_V_β_6_ negative PCa cell and non-tumorigenic prostate epithelial cells (33, 34). These results suggest that tumor-derived EVs can transfer surface proteins, especially integrins, and enhance migration and invasion of tumor cells.

EVs have also been reported to be involved in PCa drug resistance (36). Panagopoulos and co-workers (37) showed increased camptothecin (CPT, chemotherapy drug) resistance in CPT sensitive DU145 cells when EVs isolated from CPT resistant RC1 PCa cell line conditioned growth media was applied. However, when RC1 cells were cultured with EVs isolated from DU145, CPT-induced apoptosis was increased in the RC1 cells. In addition, when PCa cell lines DU-145 and 22Rv1 cells were cultured with the presence of EVs isolated from docetaxel resistance, PCa cell lines demonstrated docetaxel resistance (38). Furthermore, application of exosomes isolated from docetaxel responding PCa patients (n=6) and non-responding PCa patients (n=2) to the DU145 PCa cell line showed increased docetaxel-resistance in DU145 cells cultured with exosomes isolated from docetaxel non-responders, suggesting the potential role of tumor-cell derived EVs in drug resistance (38).

## Exercise and Prostate Cancer

Multiple epidemiological studies in clinical oncology have consistently reported the positive impact of exercise in reducing PCa progression and enhancing survival. For instance, Kenfield and co-workers reported a 61% reduced risk (P=0.03) of PCa-related death (6) while Richman and co-workers reported a 57% reduction in disease progression (P=0.03) (7) in those with higher physical activity levels (≥3 hours/week) compared to those with lower physical activity levels. Furthermore, in preclinical studies, direct application of human serum obtained after exercise from healthy individuals to PCa cell line LNCaP showed a significant reduction in LNCaP cell growth (12). In addition, reduced tumor volume and delayed tumor growth was evident in a murine model injected with LNCaP cells exposed to human serum obtained after a bout of exercise compared with cells exposed to human serum acquired before exercise, suggesting a potential role for exercise in reducing tumor progression *in vivo* (12). Similar results were shown in the report by Hwang et al. (11), where human serum obtained after exercise from healthy older individuals (age > 60) was directly applied to PC-3 PCa cell lines. In addition, exercise increased blood delivery at the tumor site in a PCa animal model (R-3327 MatLyLu tumor cell orthotopically injected mice model) and reduced aggressiveness of PCa cells *via* reduced hypoxia at the tumor site (39) suggesting that exercise-induced physiological changes might have positive effects on cancer progression.

In addition, increased lean mass in PCa patients might also positively impact patient outcomes (10). In our recent systemic review and meta-analysis of the efficacy of exercise in improving supportive care outcomes in PCa patients with a range of treatments, improvements (P<0.001) in whole-body fat mass (-0.6 kg), lean mass (+0.5 kg), and appendicular lean mass (+0.4 kg) after exercise compared to usual care were noted (9). Furthermore, in our randomized controlled trial involving 57 PCa patients undergoing androgen deprivation therapy (ADT), 3 months of exercise significantly increased muscle strength, physical function, lean mass, and a number of patient-reported outcomes (8). Moreover, a recent retrospective report also showed that men with PCa exhibited increased prostate-specific antigen (PSA) progression-free survival and radiological progression-free survival in those with higher lean mass (P=0.03 and P<0.001, respectively) (10), providing a strong case for PCa patients to engage in exercise, especially of an anabolic nature to enhance or preserve lean mass.

## Skeletal Muscle Derived EVs During Exercise and Prostate Cancer

**Figure 2:**
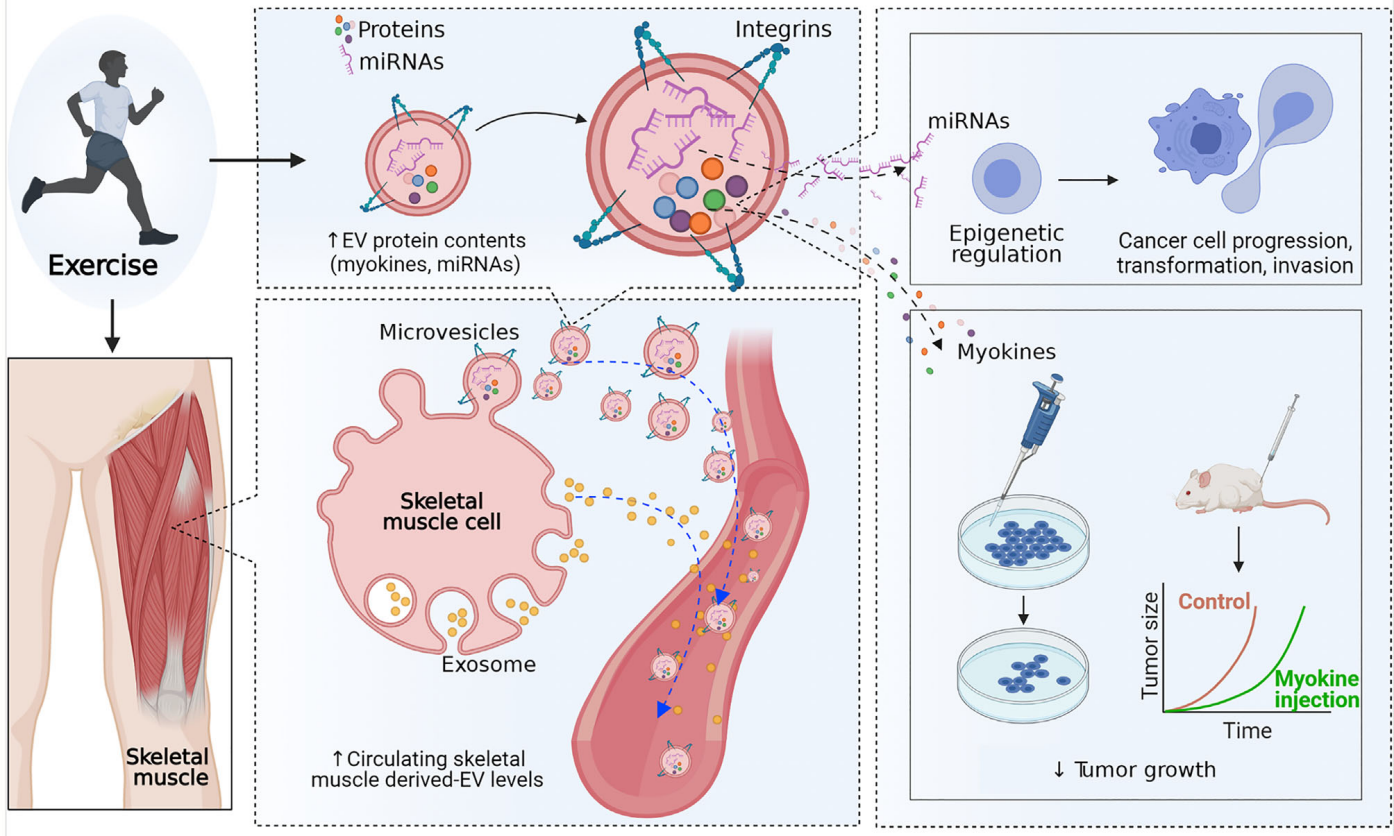
Effect of exercise on extracellular vesicles. Exercise may increase the secretion of extracellular vesicles (EVs) into the circulatory system and alter the concentration of proteins and microRNAs (miRNAs) in skeletal muscle-derived extracellular vesicles. MicroRNAs in skeletal muscle-derived extracellular vesicles may induce epigenetic regulation in prostate cancer cells and reduce cell progression, transformation, and invasion. In addition, skeletal muscle-derived extracellular vesicles containing proteins, including myokines, have a direct tumor-suppressive effect.

Over the past decade, skeletal muscle has been identified as an important secretory organ producing a range of cytokines and peptides called myokines (40). In addition, in response to exercise training, skeletal muscle releases miRNAs into the circulation (41), and miRNAs, messenger RNAs (mRNAs), DNA, piwi-interacting RNAs (piRNAs), transfer RNAs (tRNAs), and myokines loaded into EVs for intercellular communication (20, 21). Moreover, clinical and preclinical studies have shown alteration of skeletal muscle-derived EVs concentration in the circulatory system and their contained protein levels are also altered by exercise (21, 42–48) (**Table 1** and **Figure 2**).

**Table 1:** Effect of exercise on circulating extracellular vesicle concentration and contents.

Ref.	Subject	Subject number	Exercise protocol	Results
Frühbeis et al. (44)	Healthy human (male)	*Cycling*; n=8	Incremental cycling (increase power by 50 W every 3-min until exhaustion)	*Cycling*: 2.7 fold ↑ in small EVs; Flot1, Hsp/Hsc70, Tsg101 ↑ (average 5.2 fold)
		*Treadmill*; n=4	Incremental treadmill (increase speed by 2 km/h every 3-min until exhaustion)	*Treadmill*: 1.5 fold ↑ in small EVs; significant ↑ Flot 1
Oliveira et al. (47)	Rats	*Non-ex*; n=4	Acute aerobic exercise	Serum EVs concentration ↑ (non-ex, 1.1x10^9^ unit/ml; low-ex, 3x10^9^ unit/ml; mod-ex, 2.5x10^9^ unit/ml; high-ex, 3.0x10^9^ unit/ml); EVs protein concentration ↑ (non-ex, 0.935 mg/ml; low-ex, 4.33 mg/ml; mod-ex, 4.31 mg/ml; high-ex, 4.31 mg/ml); rno-miR330-5p, 10b-5p, 142-3p, and 410-3p ↑in exercise-EVs
		*Low-ex*; n=5	(40 min)	
		*Mod-ex*; n=4	*Low-ex*: 14-16 m/min	
		*High-ex*; n=5	*Mod-ex*: 20-22 m/min	
			*High-ex*: 24-26 m/min	
Bei et al. (42)	Human,	*Human*; n=16	*Human*: acute exercise stress test till exhaustion	*Human*: EVs ↑ at peak exercise compared to rest
	Mice	*Mice*; n=4	*Mice*: 5-90 min swimming, 2 day/week for 3 weeks	*Mice*: 1.85-fold increase after 3-week swimming exercise
Bertoldi et al. (43)	Rat	*3-m-old*; n=12 (ex=6, con=6)	Daily 20 min treadmill running for 2 weeks	CD36 (exosome marker) ↑ after 18 hours following exercise cessation compared to the control group
		*21-m-old*; n=12 (ex=6, con=6)		
		*26-m-old*; n=10 (ex=5, con=5)		
Nielsen et al. (46)	Healthy human, T2DM patients	*Healthy subjects*; n=14	Acute aerobic exercise (60min) 70% VO_2_max	*Healthy subjects*: 52%↑CD36^+^ SkM-EVs after exercise
		*T2DM patients*; n=13		*T2DM patients*: 55% ↑CD36^+^ SkM-EVs after exercise
				53% ↑FATP4^+^SkM-EVs after exercise
Rigamonti et al. (48)	Obese human, Healthy human	*Obese subject*; n=15	Acute moderate constant workload exercise (30 min) 60% VO_2_max	SCGA^+^EVs ↑ immediately after exercise, CD14^+^EVs ↔, CD62^+^EVs ↔, FABP^+^EVs ↔
		*Healthy subject*; n=8		
Whitham et al. (21)	Healthy human	n=22	Acute cycling until exhaustion (~60 min)	Circulating small vesicle levels ↑ immediately after exercise, 322 EV protein contents altered
			30 min at 55% VO_2_max, 20 min at 70% VO_2_max, ~10 min (until exhaustion) at 80% VO_2_max	

EV, Extracellular vesicle; Non-ex, non-exercise group; Low-ex, low-intensity exercise group; Mod-ex, moderate-intensity exercise group; High-ex, high-intensity exercise group; T2DM, type II diabetes; SkM-EVs, skeletal muscle-derived extracellular vesicle; FATP4, long-chain fatty acid transport protein 4; SCGA, sarcoglycan-α; FABP, fatty acid binding protein. ↑ indicate significant increase (p<0.05); ↔ indicate no change.

### Effect of an Exercise Bout on Skeletal Muscle-Derived EVs

Emerging clinical exercise trials are building our understanding of the effect of exercise bouts on exercise-derived EVs in both human and animal models (**Table 1** and **Figure 2**). Frühbeis and colleagues (44) showed that the level of small EVs of 100-130 nm that carry proteins characteristic of exosomes in plasma increased by an average of 5.2 times (Flot1, *P*=0.0021; Hsp/Hsc70, *P*=0.0021) in 12 healthy individuals in response to cycling or running until exhaustion. Similarly, another acute exhaustive exercise trial involving 16 healthy subjects also demonstrated a significant increase of EVs in serum at the peak exercise workload compared to at rest (*P*<0.05) (42), indicating an effect of exhaustive exercise in increasing circulating EVs concentration.

Elevation of serum EV concentrations and protein contents have also been shown after moderate-intensity aerobic exercise. Nielsen and colleagues demonstrated elevation of CD36^+^ and FATP4^+^ skeletal muscle-derived EVs in serum after 60 minutes of moderate-intensity aerobic exercise (70% VO_2_max) in both healthy (n=14; CD36^+^, 52%, *P*=0.019) and patients with metabolic disease (n=13; CD36^+^, 55%, *P*=0.016; FATP4^+^, 53%, *P*=0.007) compared to at rest (46). Moreover, Rigamonti and colleagues (48) showed a significant elevation of skeletal muscle-derived EVs (sarcoglycan-α^+^EVs) in serum obtained immediately after 30 minutes of aerobic exercise (60% VO_2_max) compared to at rest (*P*=0.016), whereas monocyte/macrophage (CD14^+^EVs), endothelium (CD62E^+^EVs), and adipose tissue (FABP^+^EVs) derived EVs were unchanged. In addition, a study investigating the effect of 60 minutes of cycling exercise on circulating small vesicle concentrations and contents in 11 healthy subjects showed elevated circulating small vesicle levels with a significant alteration of 322 proteins after the exercise bout compared to pre-exercise (21).

Not only moderate-intensity aerobic exercise but high and low-intensity exercise also have been shown to increase serum EVs concentration and EVs protein content (45, 47). A clinical study involving a 40-minute bout of vigorous-intensity aerobic exercise (80%VO_2_ max) in 22 healthy subjects reported the presence of skeletal muscle-derived EVs (sarcoglycan-α^+^EV) in a cytofluorimetric analysis and level of muscle-specific mRNAs, such as miR-181a-5p and mi-133b, in sarcoglycan-α^+^EVs to be significantly increased after exercise (*P*<0.05) (45). Furthermore, in a preclinical study, 18 mice were divided into 4 groups, non-exercise, low-intensity exercise, moderate-intensity exercise, and high-intensity exercise, and undertook 40 minutes of treadmill exercise at a speed of 14-16 m/min (20% below maximum lactate steady state (MLSS), 20-22 m/min (at MLSS), and 24-26 m/min (20% above MLSS) (47). Although there were no significant differences among exercise groups in serum EVs concentration, a significant difference in EVs concentration was shown in all exercise groups compared to the non-exercise group (low-ex *vs*. non-ex, *P*=0.014; mod-ex *vs*. non-ex, *P*=0.021; high-ex *vs*. non-ex, *P*=0.02) (47), whereas the size of EVs in the exercise groups was not changed compared to the non-exercise group. Similarly, EV protein concentrations were also significantly increased in all exercise groups compared to the non-exercise group (*P*=0.014) (47). In addition, 12 miRNAs in serum EVs (rno-miR-128-3p, 1033p, 330-5p, 148a-3p, 191a-5p, 10b-5p, 93-5p, 25-3p, 142-5p, 3068-3p, 142-3p, and 410-3p), predicted to target genes involved in the MAPK signal transduction pathway, were found to be differentially expressed after exercise in the animal model (47). These results suggest that low- and high-intensity exercise may also increase EV concentrations and protein content levels.

### Effect of Chronic Exercise Training on Skeletal Muscle-Derived EVs

Due to the lack of clinical studies investigating the effect of chronic exercise training on circulating EVs concentration, insight into the effect of exercise training on resting circulating EVs concentration can only be derived from animal studies. However, positive associations between aerobic capacity (VO_2_max) and EVs containing miRNAs (miR-1, R=0.58, *P*=0.01; miR-133b, R=0.54, *P*=0.02; miR-181a-5p, R=0.63, *P*=0.006; miR-206, R=0.5, *P*=0.003; miR-499, R=0.54, *P*=0.02) were found in the study by Guescini and colleagues (45) involving 18 healthy subjects suggesting that improvements in aerobic capacity due to chronic exercise might have a role in altering EV contents. Furthermore, a study by Bei and co-workers showed a 1.85-fold increase of circulating EVs after 3 weeks of swimming exercise in a mice model (42), and Bertoldi and colleagues demonstrated elevation of CD36 (exosome marker) in serum after 2 weeks of daily moderate-intensity exercise in a rat model at different ages (43). Although these studies suggest the elevation of circulating EVs concentration, the exercise period was short, and substantial exercise adaptation may not have occurred in the animals. Longer duration studies for exercise adaptation and investigation of the origin of these EV responses to chronic training are required to enhance our understanding of the effect of chronic exercise on circulating skeletal muscle-derived EV resting concentrations.

### Potential Role of Skeletal Muscle-Derived EV Cargoes in Prostate Cancer

Exercise can modify the biology of PCa *via* its effects on muscle hypertrophy, adipose tissue oxidation, increased insulin sensitivity, increased osteogenesis, reduced inflammation, and increased antitumor activity (49). Among the physiological alterations induced by exercise, the tumor-suppressive role of skeletal muscle secreted proteins (myokines) and miRNAs in PCa suggests a regulatory role of skeletal muscle-derived EV-containing proteins in PCa (**Figure 2**).

Myokines, such as IL-6, irisin/FNDC5, decorin, oncostatin M (OSM), and secreted protein acidic and rich in cysteine (SPARC), have shown the potential of a direct tumor-suppressive effect in different cancer cell lines, including PCa (13). For example, *in vitro* administration of IL-6 resulted in a reduction of hormone-sensitive PCa cell line proliferation by reducing androgen receptor expression (50), and application of irisin to PCa cell lines significantly reduced cell viability (*P*<0.05) (51). Moreover, direct application of SPARC and decorin significantly reduced PCa cell line growth by reduced Cyclin D1 and epidermal growth factor receptor (EGFR) activation, respectively (52, 53). Levels of myokines have also been shown to be altered in intercellular muscle protein and mRNA levels (13), supporting the analytic results of protein contents in skeletal muscle-derived EV contents by Whitham and co-workers, which showed alteration of protein contents in skeletal muscle-derived EVs (21).

The importance of epigenetics (including DNA methylation, histone modification, and miRNA *and* long non-coding RNA (lncRNA) regulation) in cellular transformation, tissue invasion, induction of angiogenesis, escape from immune surveillance, and metastasis is increasingly being recognized in cancer development and progression. In the pathogenesis of human PCa, somatic epigenetic alterations appear earlier than genetic changes, as well as more commonly and more consistently (54). Recent research has implicated EVs in epigenetic regulation of the cancer microenvironment to affect cancer progression (55). Bioinformatic analysis has indicated that many mRNAs and proteins contained in EVs are involved in epigenetic modulation (56). Proteins, mRNAs, microRNAs, and non-coding RNAs in EVs alter the phenotype of target cells by transferring mRNA, a transcriptional modulator, or degrading mRNA rapidly (57). Recently, it has been shown that aerobic exercise is a potential epigenetic modifier. Aerobic exercise induces epigenetic changes through several mechanisms, including chromatin methylation, histone acetylation, DNA methylation, and miR expression. MiRs secreted into the extracellular microenvironment *via* EVs may play an important role in epigenetic modulation (58).

### Uptake of Skeletal Muscle-Derived EVs by Prostate Cancer Cells: Potential Myokine Involvement

**Figure 3:**
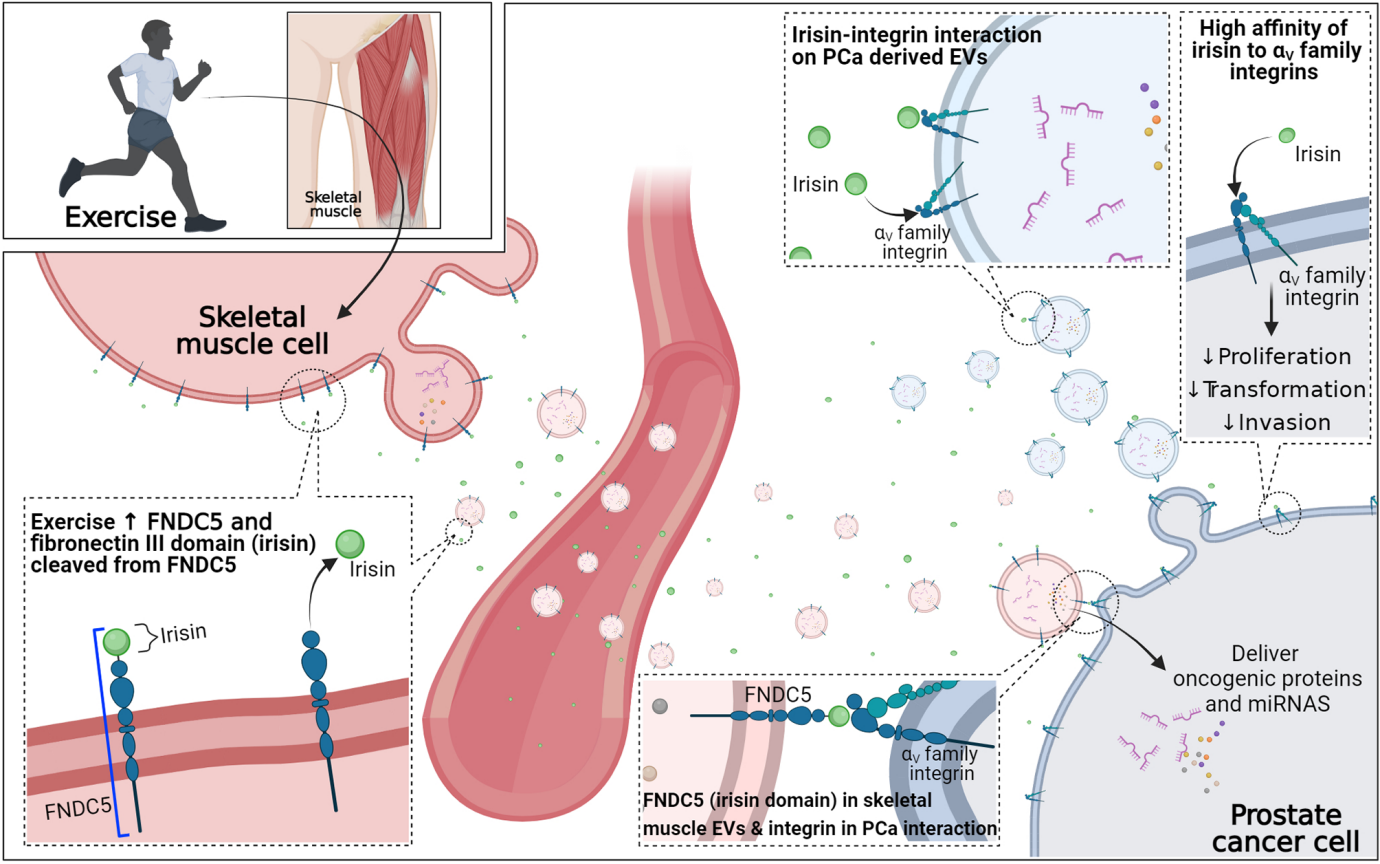
Potential role of exercise-induced myokines in extracellular uptake in prostate cancer cells. Irisin has a high affinity to α_V_ family integrins, which is highly expressed in prostate cancer. FNDC5 is a precursor of irisin and with exercise stimulation FNDC5 is increased on the membrane, as is the secretion of irisin from skeletal muscle. Free irisin and skeletal muscle-derived extracellular vesicles (EVs) containing FNDC5 may travel to the prostate cancer site through the circulatory system. The high affinity of free irisin to α_V_ family integrins on the prostate cancer cell may directly induce irisin-integrin interaction to elicit the direct tumor-suppressive effect. Moreover, free irisin may also interact with α_V_ family integrins on tumor-derived extracellular vesicles to interfere with the remodeling of metastatic niches. Lastly, the irisin domain of FNDC5 on the membrane of skeletal muscle-derived extracellular vesicles may increase internalization of skeletal muscle-derived extracellular vesicles to prostate cancer cells through FNDC5 (irisin domain)- α_V_ family integrin interaction.

Uptake of EVs by targeted cells is an important process to elicit the functional effects by initiating signaling events at the surface of recipient cells or transferring EV contents into recipient cells (59). Although the uptake of EVs by recipient cells is a critical process in cell-to-cell or ECM-to-cell communication *via* EVs, this process is poorly understood because the uptake of EVs depends on the specific properties of the recipient cells (60). However, the potential role of skeletal muscle-induced myokines, especially irisin, in skeletal muscle-derived EVs and cancer cell communication has been proposed in a recent review by Darkwah et al. (17) (**Figure 3**).

Fibronectin type III domain containing 5 (FNDC5), a type1 transmembrane glycoprotein embedded in the skeletal muscle cell membrane, is a precursor of irisin and with exercise stimulus not only does FNDC5 expression increase on the membrane of skeletal muscle cells but the fibronectin III domain is cleaved and released to the extracellular site as irisin (61, 62). Irisin has been shown to increase energy expenditure by inducing white adipocyte browning and helps to maintain metabolic homeostasis, reducing body weight, improving glucose metabolism, and improving insulin sensitivity (62–64). Furthermore, various preclinical studies have demonstrated a direct reduction of growth of various cancer cell lines after applying exogenous irisin including PCa (13).

The receptors of irisin were not identified until recently. However, irisin has been shown to have a high affinity to specific integrin families, α_V_ family, in bone and fat cells (65). Furthermore, previous studies have demonstrated high α_V_ family integrins (such as α_V_β_6_ and α_V_β_3_) expression in PCa cell lines, and these transmembrane proteins can be transferred to other recipient cells (33, 34), suggesting the potential role of irisin in increasing skeletal muscle-derived EVs and PCa cell communication. Although there is no research investigating the expression of FNDC5 in the membrane of skeletal muscle-derived EVs, the process of microvesicle biogenesis suggests the surface protein of the cell might be transferred to EVs (17). Taken together, it could be proposed that irisin-α_V_ family integrins interaction can directly induce a tumor-suppressive effect on PCa cells and possibly increase skeletal muscle-derived EVs-PCa cell communication through an extracellular domain of FNDC5 (irisin)-integrin interaction. This may increase the internalization of skeletal muscle-derived EVs into PCa cells to elicit the functional role of EV contents in PCa (17). Furthermore, circulating irisin may also interact with PCa cell-derived EV containing integrins and interfere with the delivery of PCa cell-derived EVs to the cells near PCa cells and contribute to remodeling the pre-metastatic environment (17). However, further investigation examining the role of irisin-integrin interaction in PCa is required to fully elucidate skeletal muscle-derived EVs uptake in PCa and involvement of myokines.

## Discussion

The importance of exercise oncology, the application of exercise medicine in cancer, has been well recognized in clinical oncology (5). Epidemiological (6, 7, 10) and clinical (8, 9) studies examining the effect of exercise in PCa patients have further established the role of exercise in patient care for men with PCa. Moreover, exercise-induced circulating factor alteration, epigenetic modulation, and gene expression have been suggested as potential mechanisms whereby exercise may impact disease progression in men with PCa (13, 14).

Recently, extracellular vesicles have been highlighted in cell-to-cell and cell-to-extracellular matrix communications, and in PCa tumor-derived EVs have been suggested as a therapeutic target (24–26). Tumor-derived EVs have potential in delivering oncogenic proteins, surface proteins, and miRNAs to non-tumor cells and contribute to progression of PCa by initiating growth-promoting signal cascades or creating metastatic niches among non-tumoral cells near cancerous cells (29, 31, 33, 34). As such, multiple proteins, miRNAs, and surface proteins in tumor-derived EVs have been identified as potential predictable markers for PCa progression (22, 23, 26). However, a few clinical exercise trials demonstrating elevation of skeletal muscle-induced EVs after exercise and preclinical studies demonstrating a potential tumor-suppressive effect of skeletal muscle-derived factors (such as myokines and miRNAs) suggest EVs as a potential delivery mechanism for skeletal muscle induced proteins and miRNAs in PCa (13, 21, 42–48, 58). Furthermore, the potential role of myokines in the facilitation of skeletal muscle-derived EV uptake in cancer has been recently proposed in a review article by Darkwah and co-workers (17).

It is important to note that research into skeletal muscle-derived EVs in cancer is at an early stage; more research is required to fully elucidate the role of exercise in PCa. For instance, the clinical exercise trials investigating the alteration of skeletal muscle-derived EVs after exercise are limited to healthy populations, limiting the generalizability of these outcomes to cancer patients. Moreover, as common adverse effects of ADT, a widspread treatment in men with PCa, are a significant loss of skeletal muscle mass and gain of fat mass (4), physiology of skeletal muscle biogenesis may differ from that of healthy subjects (13). The lack of preclinical studies investigating the direct effect of skeletal muscle-derived EVs on PCa cells also prevents a clear understanding of the role of exercise-induced skeletal muscle-derived EVs. Although skeletal muscle secretomes, such as myokines and miRNAs, have been shown to have potential in suppressing tumor growth (13, 49), the direct communication between skeletal muscle cells and PCa cells *via* EVs is yet to be reported.

## Conclusions

Exercise-derived EVs have received increased attention as they provide an opportunity to understand the mechanistic benefit of exercise in cancer patients. Emerging evidence indicates that aerobic exercise affects circulating EV dynamics, including the size, morphology, and composition. Proteins, miRNAs, mRNAs, and DNAs packed in exercise-specific EVs may potentially play a role in preventing PCa development and disease progression. This review has summarized the preliminary evidence for an effect of exercise on circulatory levels of skeletal muscle-derived EV secretion, EV-containing protein or miRNA contents and the role of EV-containing factors in PCa progression, as well as the potential involvement of myokines in EV uptake in PCa. Given the speculative nature of the role of exercise-derived EVs to date, in the coming decade research will likely clarify the role of EVs with a focus on the dynamics of EVs in response to specific exercise modes and dosages, providing opportunities to enhance our understanding of the tailoring of exercise prescription on mediating possible cancer outcomes.

## Author Contributions

YZ and J-SK contributed equality to this work. YZ and T-ZW drafted the initial manuscript, and J-SK developed the manuscript. J-SK, RN, DG, RG, MH, and DT performed the revision. All authors contributed to the article and approved the submitted version.

## Funding

J-SK is supported by National Health and Research Council Centre of Research Excellence (NHMRC-CRE) in Prostate Cancer Survivorship Scholarship. DG and RN are funded by an NHMRC-CRE in Prostate Cancer Survivorship (APP1116334).

## Conflict of Interest

The authors declare that the research was conducted in the absence of any commercial or financial relationships that could be construed as a potential conflict of interest.

## Publisher’s Note

All claims expressed in this article are solely those of the authors and do not necessarily represent those of their affiliated organizations, or those of the publisher, the editors and the reviewers. Any product that may be evaluated in this article, or claim that may be made by its manufacturer, is not guaranteed or endorsed by the publisher.
